# Key dimensions of women’s and their partners’ experiences of childbirth: A systematic review of reviews of qualitative studies

**DOI:** 10.1371/journal.pone.0299151

**Published:** 2024-03-29

**Authors:** Yael Benyamini, Amy Delicate, Susan Ayers, Pelin Dikmen-Yildiz, Olga Gouni, Sigridur Sia Jonsdottir, Sigfridur Inga Karlsdottir, Burcu Kömürcü Akik, Julia Leinweber, Sylvia Murphy-Tighe, Zada Pajalic, Olga Riklikiene, Claudia Maria Limmer

**Affiliations:** 1 Bob Shapell School of Social Work, Tel Aviv University, Tel Aviv, Israel; 2 Centre for Maternal and Child Health Research, City, University of London, London United Kingdom; 3 Department of Psychology, Fen—Edebiyat Fakültesi, Cumhuriyet Mahallesi, Kirklareli University, Kırklareli, Turkey; 4 Cosmoanelixis, Prenatal & Life Sciences Educational Organization, Nea Ionia, Athens, Greece; 5 School of Health Sciences, University of Akureyri, Akureyri, Iceland; 6 Department of Psychology, Faculty of Languages and History-Geography, Ankara University, Ankara, Turkey; 7 Institute of Midwifery, Charité University of Medicine, Berlin, Germany; 8 Department of Nursing & Midwifery, Health Sciences Building, University of Limerick, Ireland; 9 VID Specialized University, Oslo, Norway; 10 Faculty of Nursing, Lithuanian University of Health Sciences, Kaunas, Lithuania; 11 Department of Nursing and Management, Faculty of Business and Social Sciences, Hamburg University of Applied Sciences, Hamburg, Germany; Trinity College Dublin: The University of Dublin Trinity College, IRELAND

## Abstract

**Background:**

The World Health Organization 2018 intrapartum guideline for a positive birth experience emphasized the importance of maternal emotional and psychological well-being during pregnancy and the need for safe childbirth. Today, in many countries birth is safe, yet many women report negative and traumatic birth experiences, with adverse effects on their and their families’ well-being. Many reviews have attempted to understand the complexity of women’s and their partners’ birth experience; however, it remains unclear what the key dimensions of the birth experience are.

**Objective:**

To synthesize the information from reviews of qualitative studies on the experience of childbirth in order to identify key dimensions of women’s and their partners’ childbirth experience.

**Methods:**

Systematic database searches yielded 40 reviews, focusing either on general samples or on specific modes of birth or populations, altogether covering primary studies from over 35,000 women (and >1000 partners) in 81 countries. We appraised the reviews’ quality, extracted data and analysed it using thematic analysis.

**Findings:**

Four key dimensions of women’s and partners’ birth experience (covering ten subthemes), were identified: 1) Perceptions, including attitudes and beliefs; 2) Physical aspects, including birth environment and pain; 3) Emotional challenges; and 4) Relationships, with birth companions and interactions with healthcare professionals. In contrast with the comprehensive picture that arises from our synthesis, most reviews attended to only one or two of these dimensions.

**Conclusions:**

The identified key dimensions bring to light the complexity and multidimensionality of the birth experience. Within each dimension, pathways leading towards negative and traumatic birth experiences as well as pathways leading to positive experiences become tangible. Identifying key dimensions of the birth experience may help inform education and research in the field of birth experiences and gives guidance to practitioners and policy makers on how to promote positive birth experiences for women and their partners.

## Background

The World Health Organization [1] intrapartum guideline for a positive birth experience emphasizes the importance of maternal emotional and psychological well-being during childbirth, as well as the need for safe births. Research has shown that experiences of childbirth are complex and multidimensional [2] and can range from positive and empowering to negative or even traumatic [3]. Researchers have attempted to understand the birth experience through qualitative studies, with in-depth interviews of women who gave birth, and sometimes of their partners. Many parts of this extensive literature have already been summarized by reviews and meta-syntheses, each with specific aims. However, the number of reviews makes it difficult to extract and synthesise knowledge across all of them. This led us to conduct the current review of reviews, *aimed at synthesizing the findings from reviews of qualitative research on various aspects of the childbirth experience*, *in order to identify key dimensions of this experience and provide a comprehensive overview of women and their partners’ experience*.

Studies suggest that between one third and one half of women experienced birth as positive [4, 5], and between a fifth and close to half of birthing women reported experiencing childbirth as negative or traumatic [6–9]. Negative birth experiences are particularly important because of their potential impact on women and their families [10, 11]. The bulk of research in this area has attempted to understand negative and traumatic birth experiences, focusing on their nature, assessment, risk and protective factors. A review of the predictors and outcomes of the childbirth experience identified the dearth of literature concerning positive, or even neutral, birth experiences, with greater focus on identifying risk factors for post-traumatic stress disorder (or stress symptoms) following childbirth [3], These factors may differ from those that influence the (positive or negative) appraisal of the birth experience. Therefore, in the current review *we aimed to include a range of birth experiences*, *including positive and negative*.

Childbirth is generally considered to fall under a medical or a social model [12]. This dichotomy simplifies the great variation in births, which can range from a birth in a home setting with minimal intervention to an obstetric unit with varying degrees of intervention. This continuum is related to the birth experience, with more negative experiences reported by women who have had more medical procedures, such as epidural analgesia, induction, augmentation of first stage labour, instrumental vaginal delivery and emergency caesarean section [13]. In light of this great diversity, *we reviewed reviews of the birth experience reported by general samples as well as those that focussed on specific modes and places of birth (e*.*g*., *the experience of home birth*, *caesarean section*, *induction)*.

### Defining "the birth experience"

The term ’childbirth experience’ is used to describe a woman’s and her partner’s subjective experiences of labour and birth. Some studies narrowly focussed on the ’gist’ of the birth experience as positive or negative [e.g., 14] whereas others focus on specific aspects, such as perceived control and satisfaction with support and care during birth [e.g., 15], reported levels of pain [16] and fear during birth [17], and fulfillment of expectations [18].

The variety of terms and foci makes it difficult to assess the birth experience or satisfaction with it, as can be seen by the large number of instruments developed to assess the birth experience [19] and satisfaction with care during birth [20, 21]. This variety of definitions and instruments also makes it difficult to compare findings between studies and aggregate the results in a meaningful way.

A concept analysis by Larkin, Begley (2) defined the birth experience as an "individual life event, incorporating interrelated subjective psychological and physiological processes, influenced by social, environmental, organisational and policy contexts" (p.49). Noting that the childbirth experience can be positive or negative [22], there has been attempts to define such experiences. The World Health Organization [1] defined a positive childbirth experience "as one that fulfils or exceeds a woman’s prior personal and sociocultural beliefs and expectations, including giving birth to a healthy baby in a clinically and psychologically safe environment with continuity of practical and emotional support from a birth companion(s) and kind, technically competent clinical staff" (p.1).

A recently developed woman-centred definition views a positive childbirth experience as referring “to a woman’s experience of interactions and events directly related to childbirth that made her feel supported, in control, safe, and respected; a positive childbirth can make women feel joy, confident, and/or accomplished and may have short and/or long-term positive impacts on a woman’s psychosocial well-being” [23, p.1]. Similarly, a recent woman-centred definition views a traumatic birth experience as referring “to a woman’s experience of interaction and/or events directly related to childbirth that caused overwhelming distressing emotions and reactions; leading to short and/or long-term negative impacts on women’s health and well-being" [24, p.1]. These definitions of positive or of traumatic birth experiences are very helpful for studies and interventions aimed at these experiences. However, they narrow the focus and thus do not encompass the entire body of research on birth experiences, including the many qualitative studies that uncover the full array of birth experiences.

### The complexity of the birth experience

Not all births can be placed on a positive-to-negative continuum. In fact, viewing childbirth along such a continuum may over-simplify what is known about this experience. Studies have also suggested that negative and positive elements of the birth experience can co-exist [25], and that similar conditions can lead some women to experience birth as negative or traumatic while others would view it very differently [26], further contributing to the complexity of this experience. Two recent reviews of the predictors of the childbirth experience highlight significant contradictory evidence of what influences birth experiences [3, 22], further supporting the need for the current review of reviews.

Regardless of where and how birth takes place, it is unpredictable, full of uncertainty, affected by a myriad of physical and psychological factors. These factors include women’s background and personality [27, 28], their prenatal expectations [29], as well as support and care during birth [30, 31] and other aspects of the birth experience itself. Larkin et al. [2] identified four main attributes of the birth experience: (1) it is *individual*, a special and unique experience; (2) it is *complex* and multi-dimensional; (3) it is experienced as a sequential physiological and psychological *process*; and, (4) it is a pivotal, transformative *life event*, which can deeply impact women’s life and identity. The nature of these attributes underscores the dominance of subjective psychological aspects in women’s experience of childbirth, over more objective ones such as the place and mode of birth, interventions used, and complications during birth [32, 33].

### Why is it important to understand the birth experience?

Research has consistently shown that the birth experience can have an impact on the health and well-being of women and their families. Childbirth has been described as transformative for women [34]. A positive birth experience was found to contribute to women’s self-confidence and self-esteem [34, 35] and related to favourable maternal caregiving attitudes and behaviours, while negative experiences have been found to interfere with women’s ability to bond and care for their baby [36]. Negative birth experiences have been found to lead to post-partum depression [11] and post-traumatic symptoms [37] and to affect maternal-infant attachment, women’s and their families’ mental health and their relationships even beyond the immediate childbearing period [38, 39]. Postpartum depressive and/or post-traumatic symptoms can further contribute to reduced breastfeeding [40], and poor maternal-infant attachment [41], which have been linked to infant mental health problems, child development and behavioural difficulties [42, 43]. Additionally, they can strain the couple’s relationship [44], which in turn can also affect children’s well-being. Another outcome of a negative birth experience that can change families’ lives is its effect on future reproductive plans [45]. There is increasing evidence of long-term impacts on the child’s social and cognitive development and behavioural problems, reaching into adolescence [46]. Moreover, perinatal depression and anxiety, often related to negative birth experiences, entail substantial societal costs, as shown by estimates of the lifetime health costs for the woman and the child [47].

### The rationale for the current review

Throughout history the prime concern in childbirth was the survival of the mother and baby. Today, in many countries birth is safe, yet, as noted above, many women report negative and traumatic birth experiences, which have a negative impact on their and their families’ well-being. The potential of positive birth experiences is under-acknowledged and poorly understood [34]. To be able to support women’s rights in achieving a positive birth, it is important that women and their partners, researchers, and healthcare professionals involved in the provision of maternity services understand what constitutes the childbirth experience [3]. Valid assessment of this phenomenon is only possible if we have the best understanding of the dimensions of women’s and partners’ birth experiences. These dimensions appear in hundreds of qualitative studies that provide detailed information on various aspects of childbirth, as experienced by women, many of them published in the past decade. Qualitative research provides a rich picture of the complexity of the childbirth experience. Different aspects of this extensive literature have been summarized by meta-syntheses of qualitative studies of the birth experience. While most of these reviews focused on the women giving birth, some also related to partners’ experience. We decided to include information on partners’ experience, where available, as their experiences are also important and can affect women and the couple as a whole.

The aim of the current review of reviews was *to identify key dimensions of women and partners’ childbirth experiences in order to* improve the understanding and assessment of childbirth experiences and guide interventions aimed at promoting a positive birth experience.

The following specific objectives led this review:

- *To summarize the findings from reviews of qualitative research on various aspects of the childbirth experience*, *in order to provide a comprehensive picture of this experience*, *as reported by women and their partners*
- *to include a range of birth experiences*, *positive and negative*.
- *to review reviews of the birth experience reported by general samples as well as those focussed on specific modes and places of birth (e*.*g*., *home birth*, *caesarean section*, *induction)*.
- *to identify key dimensions of women’s and their partners’ childbirth experiences by synthesizing the information from the many reviews of qualitative studies on the birth experience*.

## Methods

The protocol for this review was registered on Prospero (CRD42020189964) [48].

### Search methods for identification of studies

Systematic searches of the four electronic databases PubMed, Scopus, CINAHL and PsycINFO were conducted from June to August 2020 and updated January 2023. Databases were searched using both controlled vocabulary words and synonymous free text words "birth", “experience”, and “review”. These search terms had to be in the title/abstract of the article and were used to capture as many articles as possible. The search strategies were adjusted for the syntax appropriate for each database/platform (see S1 Table). Searches were not limited by date. The search strategy was developed by a group of six co-authors. The database searches were conducted by three co-authors (OR, CL and JL). A software package was not used for the initial search and selection processes but Rayyan was used to update the searches in January 2023.

### Eligibility criteria for selecting studies

For the purpose of the current review, we defined inclusion and exclusion criteria, which are presented in Table 1.

**Table 1:** Inclusion and exclusion criteria.

Inclusion Criteria	Exclusion Criteria
Systematically reviewed qualitative evidence	Papers which were not a systematic review
Women’s and/or men’s/partners’ experiences during labour and birth of their baby	Did not include qualitative research
Full text available in English	Non-English publications
	Conference abstracts
	Articles not including the target population (i.e. women and/or men who have experienced the birth of a baby) or did not report on women’s and/or men’s (father/partner) lived experience of birth (e.g., studies on birth expectations and not experiences; studies on interventions).

### Study selection process

**Fig 1:**
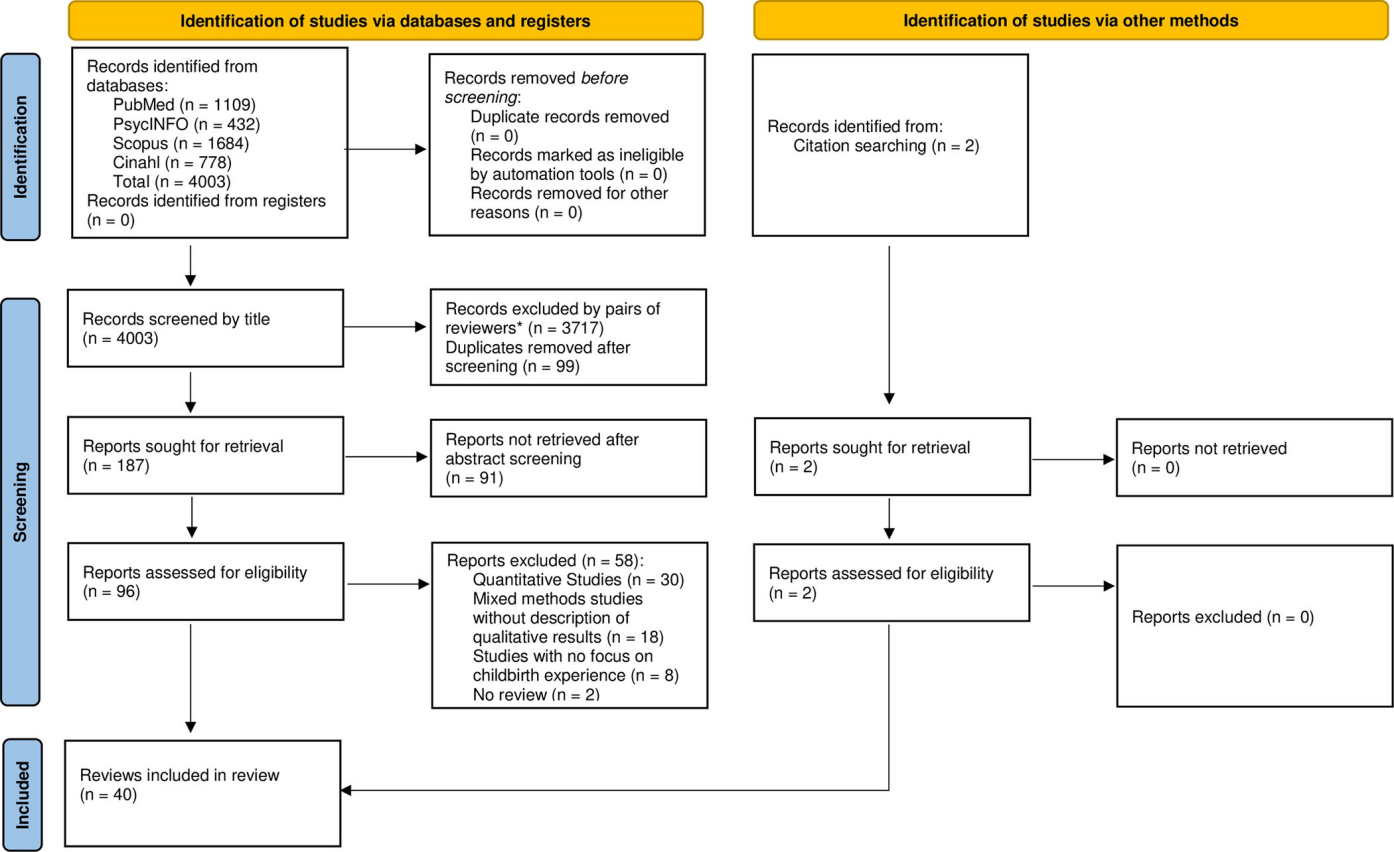
PRISMA flowchart.

The search identified 4003 records: 1109 from PubMed, 432 from PsycINFO, 1684 from Scopus and 778 from CINAHL. Two studies were retrieved from citation searching. Screening was conducted by five groups of two authors independently reviewing titles and abstracts, followed by evaluation of full text articles to determine eligibility according to the pre-defined criteria. Consensus of agreement within each pair of co-authors was met at each step of the screening process. When disagreement occurred, a third co-author was consulted. Altogether, 12 authors were involved in this step (five two-person teams; one replaced another along the process and one (CL) coordinated the work). Following initial screening, the majority of titles were excluded as they did not meet the inclusion criteria and 187 abstracts remained for further review. See Fig 1 for PRISMA flowchart of the selection process.

### Study quality appraisal

The methodological quality of the final set of 40 review articles was evaluated using the *ENhancing TRansparency in REporting the synthesis of Qualitative research* (ENTREQ) statement guidelines [49]. Each review was independently assessed by two co-authors (in the same five teams as described above) on all 21 ENTREQ items, resulting in a score of up to 21. We added two items, which were not included in the summary score: Whether the review reported any pre-registration procedure and whether they used PRISMA or another flowchart that described the search and screening results. Each pair of researchers compared their ratings and reached an agreement, consulting with a third author if needed. Studies were not excluded based on ENTREQ assessment, as the aim was to describe and synthesize qualitative literature and not to determine an effect size.

### Data extraction and management

Extraction of the characteristics of each review and the studies it included, its methodology, and main findings was also conducted in pairs of co-authors. For each review, one person extracted the information and the other reviewed the extraction for accuracy. When necessary, disagreements were resolved by consulting a third researcher. Finally, because extraction was split among five pairs, each column was reviewed in full by one co-author to ensure standardization of extraction across columns. A final review of all tables was conducted by one co-author (YB).

### Data analysis and synthesis

A thematic analysis was conducted by one co-author (AD) in consultation with another co-author (SA). All 40 reviews were read and then imported as full manuscripts into NVivo12 software for data management. For each review, themes and sub-themes relevant to the current study aims were coded as concepts in NVivo. Following the inductive process of thematic analysis, the concepts were reviewed and where similarity was identified, concepts were combined. These core concepts were compared and grouped by topic, resulting in sub-themes arranged under four main dimensions (or higher order themes). Each sub-theme and overarching dimension was given a label and description.

Next, we used the GRADE-CERQual (*Confidence in the Evidence from Reviews of Qualitative research*) framework to assess our confidence in each finding [50]. CERQual provides “a systematic and transparent framework for assessing confidence in individual review findings, based on consideration of four components: (1) methodological limitations, (2) coherence, (3) adequacy of data, and (4) relevance” (p.1). Methodological limitations were minimal because most reviews included in the current study received a high ENTREQ score and relevance was ensured by strict adherence to the inclusion and exclusion criteria. Therefore, while all four components were considered, our assessment of confidence focussed primarily on coherence, defined as “how clear and cogent the fit is between the data from the primary studies and a review finding that synthesises that data” [p.33; 51], and on adequacy, defined as “an overall determination of the degree of richness and the quantity of data supporting a review finding” [p.43; 52].

The data synthesis and the CERQual assessments were then independently appraised by four co-authors (IK, JL, OR, YB). After reading the results of all four key dimensions, each researcher then appraised one dimension and its subthemes in detail. The analysis was checked in terms of relevance of synthesis, supporting reviews and data, appropriateness of labels and descriptions, and accuracy of the CERQUAL assessment of confidence.

Resulting comments and suggested changes to synthesis, labels, and descriptions were discussed and resolution reached as a group. The co-author who conducted the initial analysis (AD) then revised the synthesis, reporting of results, and CERQual assessments based on the group‘s decisions. All 13 co-authors reviewed the results and were given an opportunity to further comment and high level of agreement was reached. Where appropriate, comments were incorporated into the results presented in this article.

Finally, the co-authors evaluated the current review and confirmed that it meets all 21 ENTREQ items.

## Results

### Review characteristics

The characteristics of the 40 reviews are presented in Table 2. These reviews reported on between four and 81 primary studies, covering a total of 759 studies, with total sample sizes ranging from under 100 to several thousand women (a few reviews did not state the sample sizes of their reviewed studies). An overlap of 142 original studies was identified among the included reviews. Thus, our analysis is based on reviews of 617 original studies. We assume that the impact of this overlap on the synthesis of qualitative reviews is negligible. Thirteen of the reviews included studies of men/partners, with samples ranging from fewer than ten to several hundred men. Altogether, the studies included over 35,000 women and over 1,000 partners. The included reviews were published between 2008 and 2023 (62% in 2018–2023). The primary studies within the reviews were undertaken across 81 countries from all continents, with the European region having the highest representation, followed by the America(s) and Australia. Seventeen reviews included only studies of general populations from high-income countries, three included migrant women in high-income countries, two included only studies from middle- or low-income countries, and the remaining 18 studies included a mixture of studies in terms of country income level.

**Table 2:** Characteristics of the included reviews.

	Authors (Year)	No. of Studies*	Total sample size**	Search period	Countries	Analysis method	Aims & Research Question/s	ENTREQ Score
**1**	Aannestad, Herstad [53] 2020	9	656 women	1995–2018	High income: Australia, Iceland, Ireland, Norway, Sweden, UK (England, Scotland)	Meta-ethnography	• Aim: To explore women’s reflections on their experiences of labour and birth and how these were influenced by the midwifery care they received. • Review question: What is the significance of the midwife in a woman’s labour and birth experiences?	19
**2**	Akuamoah-Boateng and Spencer [54] 2018	5	65 women	Not stated	High income: Australia, Canada, Ireland, UK (England, Scotland)	Meta-synthesis	• Aim: To explore women’s experiences and perceptions of induction of labour for uncomplicated post-term pregnancy in a bid to provide a woman-centred approach to the care of women with uncomplicated post-term pregnancy.	19
**3**	Anderson, Zega [55] 2021	13	648 women	To 7/2020	High income: England, Ireland, Sweden, Taiwan; Upper middle income: Jordan, South Africa, Turkey; Lower middle income: Ghana, Iran, Pakistan	Meta-synthesis	• Aim: To identify and meta-synthesize results of qualitative studies on the needs of women cared for by midwives during childbirth in hospitals.	19
**4**	Balaam, Akerjordet [56] 2013	16	393 migrant women and 7 men	1/1996-6/2010	Migrant women in European high income countries: Greece, Ireland, Norway, Sweden, Switzerland, UK (participants were mostly from Asian, African, and Eastern European ethnicities)	Thematic synthesis	• Aim: To explore migrant women’s perceptions of their needs and experiences related to pregnancy and childbirth. • Review questions: (1) What are migrant women’s experiences of pregnancy and childbirth? And (2) What are migrant women’s health needs and how can their access to maternity services be described?	18
**5**	Beake, Chang [57] 2018	21	312 women and 263 men	1/2003-6/2016	High income: UK, Ireland, Italy, New Zealand, Norway, Sweden, USA	Meta-synthesis	• Aim: To examine evidence of women’s, labour companions’ and health professionals’ experiences of management of early labour to consider how this could be enhanced to better reflect women’s needs. • Seven specific review questions were developed (two primary and five secondary). The primary questions were: (1) What are women’s, labour companions’ and health professionals’ perceptions and experiences of early labour management, including advice and support offered, prior to confirmation of onset of active labour? (2) What are the physical and psychological care needs of women and their labour companions during early labour, prior to confirmation of onset of active labour?	20
**6**	Benza and Liamputtong [58] 2014	15	323 migrant women	To 6/2013	Migrant women in high income countries: Australia, Canada, New Zealand, Switzerland, UK, USA (participants were mostly from Asian and African ethnicities)	Meta-synthesis/ Meta-ethnography	• Aim: To identify and synthesise qualitative research studies that explore the perceptions of pregnancy, childbirth and motherhood, and lived experiences of migrant women who have settled in a western country.	19
**7**	Bohren, Vogel [59] 2015	65	>5,000 women and several dozen men	To 2/2015	59 qualitative studies included here from 32 countries: High income (some of immigrant populations): Australia, Canada, New Zealand, Sweden, UK, USA; Upper Middle income: Brazil, China, Cuba, Jordan, Serbia, South Africa, Turkey; Lower Middle income: Bangladesh, Benin, Bolivia, Cambodia, Egypt, Ghana, India, Kenya, Morocco, Nigeria, Palestinian Authority, Tanzania, Zambia; Low income: Afghanistan, Ethiopia, Malawi, Sierra Leone, Uganda	Thematic synthesis	• Aim: To synthesize qualitative and quantitative evidence on the mistreatment of women during childbirth in health facilities to inform the development of an evidence-based typology of the phenomenon. • The findings from the 59 qualitative studies were used here. The authors stated that “Most of the quantitative findings fit into the themes constructed from the qualitative synthesis” (p. 5).	20
**8**	Bohren, Berger [60] 2019	51 studies (52 articles)	Not stated (women and men)	To 9/9/2018	51 studies in 22 countries: 33 studies in high income: Australia, Sweden, Canada, Finland, UK, USA; Upper middle income: Brazil, China, Jordan, Lebanon, Mexico, South Africa; Lower middle income: Egypt, Ghana, Iran, Kenya, Nepal, Tanzania; Low income: Malawi, Rwanda, Syria, Uganda.	Thematic synthesis	.• Aim: To describe and explore the perceptions and experiences of women, partners, community members, healthcare providers and administrators, and other key stakeholders regarding labour companionship; to identify factors affecting successful implementation and sustainability of labour companionship; and to explore how the findings of this review can enhance understanding of the related Cochrane systematic review of interventions.	21
**9**	Bradley, McCourt [61] 2016	25	646 women	1990-5/2015	Upper middle income: South Africa; Lower middle income: Ghana, Kenya, Tanzania, Zambia, Zimbabwe; Low income: Ethiopia, Gambia, Malawi	Meta-synthesis	• To examine what drives the dynamics of disrespectful intrapartum care and influences midwives to behave in the manner that women report. • To synthesize insights from women’s experiences to explore the cultural and social factors which underpin midwives’ behaviour, and to understand the dynamics at play when disrespectful care occurs.	21
**10**	Chimwaza, Kabuluzi [62] 2015	4 (5 articles)	58 men	to 11/2013	Upper middle income: South Africa; Lower middle income: Nepal; Low income: Gambia, Malawi	Descriptive (thematic) analysis	• To provide a descriptive review of qualitative studies on experiences of men who supported their partners during labour and birth in low-resource settings.	14
**11**	Clews, Church [63] 2020	5	346 women	2003–2018	High income: Australia, New Zealand, Sweden, Taiwan, USA	Meta-synthesis /Meta-ethnography	• To undertake a review of qualitative studies exploring women’s experiences of waterbirth.	17
**12**	Coates, Cupples [64] 2019	10 (11 articles)	157 women	2010–2018	High income: Australia, Ireland, UK (England, Scotland, Wales), USA; Upper Middle income: Brasil	Thematic synthesis	• To gather, analyse and synthesise the evidence on women’s views on induction of labour and understand the factors that make the process and method acceptable and positive to women, or not.	18
**13**	Coates, Thirukumar [65] 2020	26	>10,000 women (>800 in qualitative studies)	2008–2018	High income: Australia, Austria, Belgium, Canada, Germany, Israel, Japan, Sweden, UK, USA; Upper middle income: Turkey; Lower middle income: Iran, Nigeria.	Integrative synthesis	• To gain insight into women’s experiences of and satisfaction with cesarean birth (emergency or planned; medically indicated or maternally requested), and to identify factors that contribute to women’s poor experiences of care. • The review included qualitative, quantitative, and mixed-methods studies and all the findings were integrated, regardless of study design.	18
**14**	Crawford, Hayes [66] 2017	5	120 women	2008–2013	High Income: France, Germany, Italy, UK	Integrative synthesis	• To explore available evidence on women’s experiences of continuous fetal monitoring: to investigate its acceptability before clinical implementation and to inform clinical studies; and, to explore whether it has a positive or negative effect on anxiety. • Included qualitative, quantitative, and mixed-methods studies; “Qualitative and quantitative data were synthesized together to assess for agreement or disagreement” (p. 1407).	19
**15**	Crookall, Fowler [67] 2018	34	136 women (in the qualitative/mixed studies)	To 5/2017	12 qualitative studies included here (of 34): High Income: Australia, Netherlands, UK	Hybrid deductive- inductive thematic synthesis	• Aim: To explore the quantitative/qualitative literature on women’s experiences of perineal trauma sustained during childbirth and the impact it may have on psychological/ emotional well-being. • The analysis based on the 11 qualitative and 1 mixed-methods study was reported separately and used here.	18
**16**	Crossland, Kingdon [68] 2020	6	73 women and 20 men	To 04/2019	6 of 42 studies included here from: High income: Sweden, UK, USA	Meta-ethnography and narrative synthesis	• Aim: To improve understanding of the limitations, barriers and potential facilitating factors for the appropriate use of assisted vaginal delivery (AVD), from the point of view of women, service providers, policy makers, and funders. • The review questions were: (1) What views, beliefs, concerns and experiences have been reported in relation to AVD? (2) What are the influencing factors (barriers) associated with low use of/acceptance of AVD? (3) What are the enabling factors associated with increased appropriate use of/acceptance of AVD? • The analysis based on the 6 qualitative studies was reported separately and used here.	20
**17**	Deys, Wilson [69] 2021	13	230 women 21 couples43 companions and staff (in the qualitative/mixed studies)	2010–2020	High income: Australia, Canada, Germany, USA; Upper middle income: Brazil; Lower middle income: Iran	Thematic synthesis	• Aim: To synthesise original research that explores the experience of women having immediate and uninterrupted skin-to-skin contact at caesarean section when woman and baby are well. The focus was on the experience of women (even when partners and staff were included). • The analysis was based on all studies (5 qualitative, 4 mixed-methods, 4 quantitative).	18
**18**	Elmir, Schmied [70] 2010	10	267 women, 6 men	1/1994-10/2009	High income: Australia, Canada, New Zealand, UK, USA	Meta-ethnography	• Aim: To describe women’s perceptions and experiences of a traumatic birth, and to present the findings a meta-ethnographic study reporting women’s perceptions and experiences of traumatic birth.	18
**19**	Elmir and Schmied [71] 2016	8	100 men	1/2000-2/2015	High income: Japan, New Zealand, Sweden, UK	Meta-Ethnography	• Aim: To report on the findings of a meta-ethnographic synthesis of fathers’ experience of complicated births that are potentially traumatic.	20
**20**	Eri, Bondas [72] 2015	11	248 women and 49 partners	Not stated	High income: Norway, Sweden, UK (Scotland, Wales, England), USA	Meta-Ethnography	• Aim: To integrate findings of individual studies in order to broaden the understanding of first-time mothers’ experiences of early labour.	19
**21**	Fair, Raben [73] 2020	47 (51 articles)	~1500 migrant women	2007-5/2017	Migrant women in high income countries: Czech Republic, Finland, France, Germany, Greece, Ireland, Italy, Portugal, Spain, Switzerland, Sweden, Norway, Netherlands, UK (participants were mostly from Asian, African, and Eastern European ethnicities)	Thematic synthesis	• Aim: To provide up-to-date systematic evidence on migrant women’s experiences of pregnancy, childbirth and maternity care in their destination European country.	21
**22**	Heideveld-Gerritsen, van Vulpen [74] 2021	10	257 women	To Oct 16, 2020	High income: Canada, Poland, Switzerland, USA; Lower middle income: Ghana, Vietnam	Meta-aggregation and thematic analysis	• Aim: To identify and provide an overview of reported maternity care experiences of women with physical disabilities, including sensory disabilities.	21
**23**	Hoga, Reberte Gouveia [75] 2013	15	Not stated (labor companions, mostly fathers)	1996–2011	High income: England, France, Sweden, UK, USA; Upper middle income: Brazil, South Africa	Joanna Briggs Institute-Qualitative Assessment and Review Instrument (JBI-QARI)	• Aim: To critically appraise, synthesize and present the best available evidence related to the experiences and roles played by a companion during normal labour and childbirth. • The review questions were 1) What are experiences of companions during normal labour and childbirth? 2) What roles are performed by a companion in normal labour and childbirth?	19
**24**	Johansson, Fenwick [76] 2015	8	120 men	To 6/2013	High income: England, Sweden; Lower middle income: Nepal; Low income: Malawi	Meta-synthesis	• Aim: To develop greater understanding of how expectant fathers experience their partner’s labour and the subsequent birth of their baby.	19
**25**	Keedle, Schmied [77] 2018	20	274 women interviewed, 170 phone calls, 611 online entries	2000–2016	High income: Australia, Finland, Sweden, Netherlands, UK, USA	Meta-ethnography	• Aim: To explore women’s experiences of vaginal birth after cesarean section (VBAC) across a variety of birth locations.	21
**26**	Lally, Murtagh [78] 2008	32	>5000 women (>1000 in qualitative studies)	To 2007	High income: Australia, Canada, Finland, Iceland, Ireland, Israel, Sweden, UK, USA; Upper middle income: Jordan, Lebanon.	Thematic analysis	• Aim: To systematically review the empirical literature on women’s expectations and experiences of pain and pain relief during labour, as well as their involvement in the decision-making process; to understand whether expectations were met by women’s experiences. • The review was based on 13 qualitative and 19 quantitative studies, which were all used to derive the themes “in order to provide a comprehensive integrative overview of the current evidence” (p. 2).	16
**27**	Lou, Hvidman [79] 2019	8	298 women	2007–2018	High income: Canada, Ireland, USA, UK, Australia	Thematic analysis	• Aim: To summarize the current qualitative evidence on women’s experience of postterm induction of labour.	20
**28**	Lunda, Minnie [80] 2018	12	651 women	1/2005-7/2016	High income: Canada, Sweden, USA; Upper middle income: Lebanon, Russia; Lower middle income: Egypt, Nepal; Low income: Malawi, Syria	Thematic analysis	• Aim: To integrate individual studies’ findings related to women’s views and experiences of continuous support during childbirth. • The review question: What is the best available research evidence about women’s views and experiences regarding continuous support during childbirth?	20
**29**	Mannava, Durrant [81] 2015	81	>5000 women and several hundred men	1/1990-12/2014	High income: Chile, Saudi Arabia; Upper middle income: Argentina, Brazil, China, Cuba, Dominican Republic, Guatemala, Lebanon, Mexico, South Africa, Thailand; Lower middle income: Bangladesh, Benin, Bolivia, Cambodia, Congo, Ghana, India, Indonesia, Kenya, Nicaragua, Nigeria, Pakistan, Palestinian Authority, Papua New Guinea, Philippines, Tanzania, Timor-Leste, Vietnam, Zambia, Zimbabwe; Low income: Afghanistan, Burkina Faso, Ethiopia, Gambia, Malawi, Mozambique, Niger, Sierra Leone, Uganda, Yemen.	Thematic analysis	• Aim: To identify the attitudes and behaviours of formal-sector maternal healthcare providers in low and middle income countries towards their patients; influences on these attitudes and behaviours; and their impacts. • Most of the studies included patients’ perspectives, alone or together with community and/or provider perspectives (only 4 of the 81 studies included only providers). • The thematic analysis was based on all studies (only 7 of the 81 studies were quantitative; most studies included women who had given birth, some also included pregnant women, other family members, providers and other stakeholders).	16
**30**	Miyauchi, Shishido [82] 2022	22	501 women	1990 to 2020	High income: Australia, Ireland, Japan, Netherlands, Norway, Sweden, Taiwan, UK (England), USA; Upper Middle income: Brazil, Jordan, South Africa Lower middle income: Cambodia, Ghana, Kenya, Pakistan, Tanzania Low income: Malawi, Uganda	Meta-synthesis	• Aim: To explore women’s experiences of facility based childbirth to gain insights into their perceptions of women-centred care, including humanized childbirth and respectful maternity care during intrapartum care. The focus synthesised the voices of women who had experienced physiological birth in the eligible articles.	21
**31**	Olza, Leahy-Warren [32] 2018	8	94 women	To 10/2017	High income: Australia, Iceland, New Zealand, Norway, Sweden, UK	Meta-synthesis	• Aim: To synthesise qualitative studies on women’s psychological experiences of physiological childbirth, paying attention to the imminent psychological responses that emerge during the process of labour and birth.	20
**32**	Patterson, Hollins Martin [83] 2019	14	1412 women and 6 men (242 women and 6 men in the qualitative/mixed studies)	1980 to 1/2016	High income: Australia, Belgium, Italy, New Zealand, Sweden, UK, USA	Narrative synthesis	• Aim: To review primary research regarding PTSD-PC that focussed on Quality of Provider Interaction (QPI) from the perspective of women who have developed PTSD-PC, or midwives. • The narrative synthesis was based on findings from all 14 studies: 6 quantitative, 1 mixed-methods, 7 qualitative (one of the 7 included only midwives, n = 8).	18
**33**	Puia [84] 2013	10	3721 women	2000–2010	High income: Australia, Canada, England, Finland, Japan, Scotland, USA	Meta-ethnography	• Aim: To gain a more comprehensive understanding of women’s emotional and physical needs when having a cesarean birth, including in postoperative recovery, and to guide improvements in clinical practice.	12
**34**	Sands, Evans [85] 2023	15	2562 women	To June 2021	High income: Australia, UK, USA	Thematic synthesis approach	• Aim: To investigate the impact of birth environments with a specific focus on women with complex pregnancies. This included exploring the experiences, views and evaluations of women and healthcare professionals. • The thematic analysis was based on all studies (13 of the 15 studies were qualitative or mixed-methods; 7 studies included women who had given birth (4 of them also included midwives), 8 studies included midwives or other clinicians).	20
**35**	Shakibazadeh, Namadian [86] 2018	67	Not Stated	To 2/2017	High income: Australia, Canada, Chile, Finland, Hong Kong, Iceland, Italy, Japan, Norway, Sweden, Spain, Switzerland, Taiwan, UK, USA; Upper middle income: Brazil, China, Guatemala, Lebanon, South Africa, Turkey; Lower middle income: Bangladesh, Benin, Egypt, Ghana, India, Iran, Nepal, Tanzania, West Bank and Gaza; Low income: Ethiopia, Malawi	Thematic analysis	• Aim: To develop a conceptualisation of respectful maternity care (RMC) in health facilities from the perspectives of key stakeholders (including users—women and their families, providers, administrators, and policymakers). • Most studies including women’s perspectives, alone or combined with other stakeholders.	17
**36**	Shorey and Wong [39] 2022	19	1034 women 95 partners	To 04/2020	High income: Australia Ireland, Netherlands, New Zealand, Spain, Sweden, UK, USA; Upper middle income: Serbia, South Africa, Turkey; + one study from a worldwide survey	Meta-synthesis	• Aim To explore and consolidate the traumatic childbirth experiences of parents with the ultimate aim of understanding parental needs and supporting parents based on their individual needs • Provides a consolidated understanding of the perceptions of traumatic birth experiences and impact on emotional, psychological health & relationships of parents.	20
**37**	Thomson, Feeley [87] 2019	24	1787 women 57 partners, 7 birth partners	1996–2017	High income: Australia, Canada, Denmark, Ireland, New Zealand, Sweden, UK, USA; Upper middle income: Brazil, South Africa, Turkey	Thematic analysis and meta-ethnography	• Aim: To synthesise qualitative studies on women’s views and experiences of pharmacological (epidural, opioid analgesia) and non-pharmacological (relaxation, massage techniques) pain relief options, to understand what affects women’s decisions and choices and to inform guidelines, policy, and practice.	20
**38**	Van der Gucht and Lewis [88] 2015	10	158 women	1996–2014	High income: Australia, Finland, Iceland, Ireland, Sweden, UK (England); Lower middle income: Indonesia, Iran	Thematic analysis	• Aim: To identify and analyze qualitative literature exploring women’s experiences of coping with pain during childbirth.	17
**39**	Watson, White [89] 2020	5	787 women	04/2010–04/2020	High income: Australia, Ireland, UK	Scoping review using Arksey & O’Malley framework	• Aim: To explore the experiences of women who describe their birth as psychologically traumatic and whose trauma was not reported to be primarily influenced by pre-existing high stress, obstetric or contextual complicating factors as reported in the existing literature.	11
**40**	Wigert, Nilsson [90] 2020	14	242 women	To 4/2018	High income: Australia, Finland, Norway, Sweden, USA; Lower middle income: Iran	Meta-synthesis	• Aim: To synthesize qualitative literature to deepen the understanding of women’s experiences of fear of childbirth. • Several studies included pregnant women.	20

* Several reviews included quantitative and mixed-methods studies. We note in the Research Question/s column whether they analysed them separately.

** The main focus of the included reviews were women and/or their partners. Where sample size was provided only for individual studies, we calculated the total number of women and/or partners. Providers and other stakeholders were included in a few of the reviews, as can be seen in the aims, and were not included in the total sample size.

The reviews considered a range of different aspects related to birth experience, from perspectives of women as well as partners or labour companions (only four focused solely on men). Most of the evidence on which our review was based on qualitative studies of women and men/partners. However, some reviews also included quantitative studies and some the perspectives of companions other than the partner, healthcare providers, and stakeholders. Where data from different methodologies and populations were analysed separately, we extracted only the analyses of qualitative data from women and partners. Of the 40 reviews, 13 focused on the birth experience in general, of women (n = 9), men (n = 3) or both (n = 1), with two of these focusing on physiological births and four on traumatic births. The remaining 28 reviews focused on a specific type of birth or particular aspect of the birth experience, with one to three reviews on each of the following topics: Early labour, induction of labour, assisted vaginal delivery, cesarean section, vaginal birth after cesarean section, continuous fetal monitoring, immediate skin to skin at cesarean section birth, pain or pain relief during labour, perineal trauma, waterbirth, fear of childbirth, birth environment in complex pregnancies, companionship /continuous support, post-traumatic stress disorder (PTSD) following childbirth, mistreatment or (dis)respectful intrapartum care, attitudes and behaviours of healthcare providers. One review involved the experiences of women with physical disabilities.

### Quality appraisal

Most reviews were of good to excellent quality (i.e., ENTREQ scores of 16 to 21, the highest possible score, with three exceptions of scores of 14, 12 and 11). We did not exclude any studies on the basis of their quality rating. However, we considered the study quality when assessing our confidence in own findings (with CERQual, see below). The high quality of most of the reviews along with the broad inclusion criteria used to identify them suggest that the risk of bias in the information on which this review is based is low. The summary ENTREQ scores appear in Table 2 and the detailed ratings are shown in S2 Table.

### Synthesis

**Fig 2:**
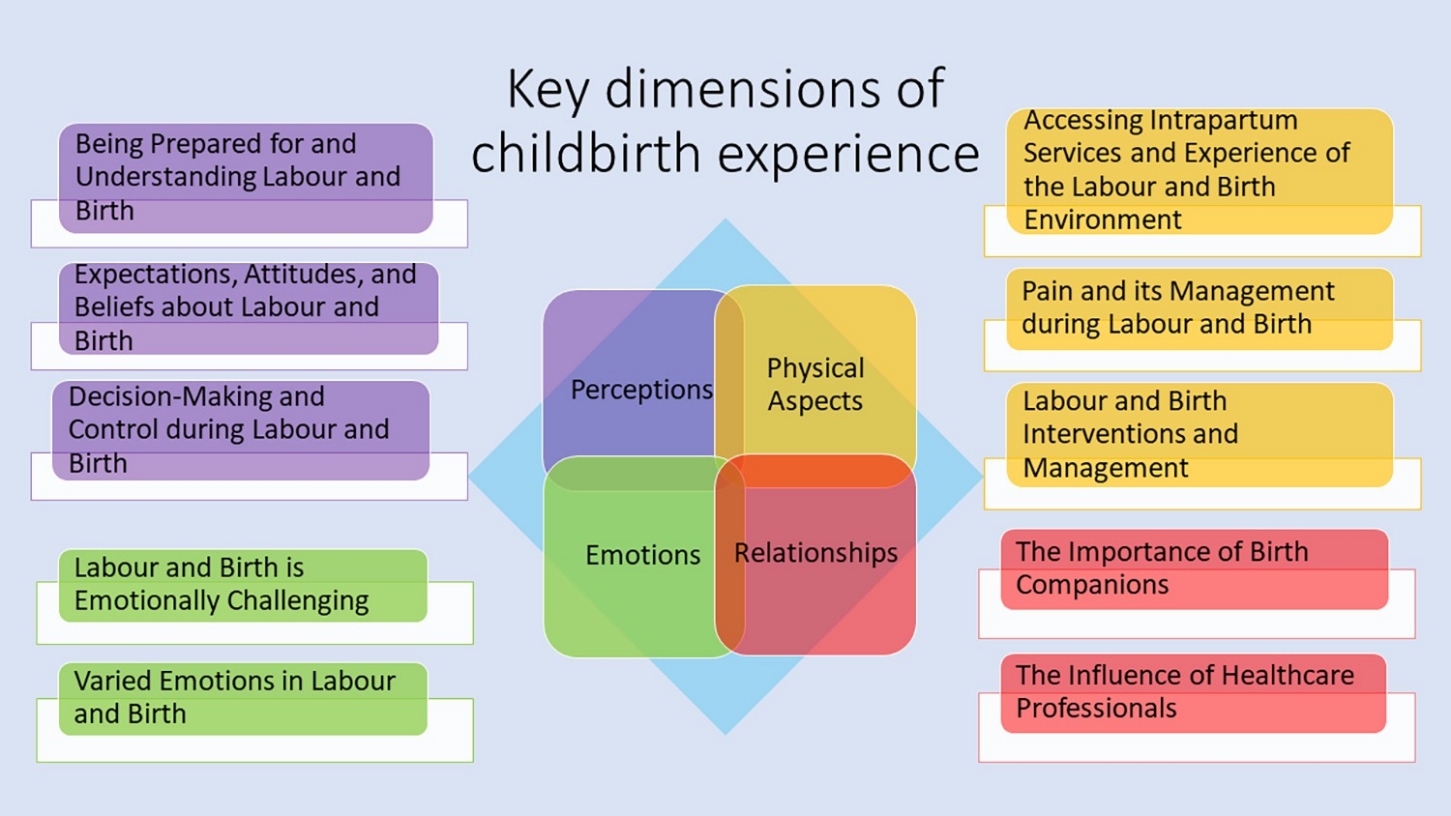
The four key dimensions of the childbirth experience and their respective subthemes.

Thematic analysis resulted in ten sub-themes supporting four dimensions of the birth experience (Fig 2): Theme 1 Perceptions; Theme 2 Physical aspects; Theme 3 Emotions; Theme 4 Relationships. Table 3 details the sub-themes and indicates which of the reviews supported each sub-theme.

**Table 3:** Reviews supporting themes.

Theme	Sub-Themes	Reviews																																							
		Aannestad et al., 2020	Akuamoah-Boateng & Spencer, 2018	Anderson et al., 2021	Balaam et al., 2013	Beake et al., 2018	Benza & Liamputtong, 2014	Bohren et al., 2015	Bohren et al., 2019	Bradley et al., 2016	Chimwaza et al.,2015	Clews et al., 2020	Coates et al., 2019	Coates et al., 2020	Crawford et al., 2017	Crookall et al., 2018	Crossland et al., 2020	Deys et al., 2021	Elmir et al., 2010	Elmir & Schmied., 2016	Eri et al., 2015	Fair et al. 2020	Heidefeld-Gerritsen et al., 2021	Hoga et al., 2011	Johansson et al., 2015	Keedle, 2018	Lally et al.,2008	Lou et al., 2019	Lunda et al., 2018	Mannava et al., 2015	Miyauchi et al., 2022	Olza et al., 2018	Patterson et al., 2019	Puia, 2013	Sands et al., 2023	Shakibazadeh et al.2017	Shorey & Wong, 2022	Thomson et al., 2019	Van der Gucht & Lewis, 2015	Watson et al., 2021	Wigert et al., 2020
1	Being Prepared for and Understanding Labour and Birth		X	X		X					X	X				X	X				X		X		X						X										X
	Expectations, Attitudes, and Beliefs about Labour and Birth		X		X	X	X		X			X						X				X						X									X	X			X
	Decision-making and Control During Labour and Birth		X	X						X		X	X					X	X								X	X					X	X		X				X	
2	Accessing Intrapartum Services and Experience of the Labour and Birth Environment			X		X					X		X								X		X							X	X				X	X					
	Pain and its Management During Labour and Birth			X								X				X											X				X	X		X			X	X	X		
	Labour and Birth Interventions and Management		X	X												X										X		X													
3	Labour and Birth as Emotionally Challenging					X	X		X		X			X	X					X	X	X			X	X					X	X		X			X				
	Varied Emotions in Labour and Birth										X				X		X								X	X					X	X									
4	The Importance of Birth Companions			X		X			X		X													X	X				X		X		X			X			X		
	The Influence of Health Care Professionals	X		X	X	X		X	X	X				X					X		X	X	X	X	X					X	X		X	X		X	X	X		X	

CERQual assessments of confidence in these findings ranged from moderate to high. Across sub-themes there were no or very minor concerns regarding methodological limitations and relevance. There were minor concerns over coherence in 6 of the 10 sub-themes due mostly to the narrow focus of some supporting reviews. Regarding adequacy, there were minor concerns in 5 of the 10 sub-themes owing to the small sample size (5 or fewer studies) in a few of the supporting reviews for these subthemes. Full details of the CERQual assessment of confidence are available in S3 Table. All steps of reviewing are further demonstrated in the PRISMA 2020 checklist (S1 Checklist).

#### Dimension 1 perceptions

A person’s prior beliefs, opinions, and attitudes toward childbirth, as well as those of the people around them, may shape childbirth expectations and experiences. Differences between expectations and the experience of labour and birth may contribute to personal interpretation of the lived experience. Awareness and understanding of the multi-faceted process of giving birth may change antenatally, during labour, or due to reflection postpartum. Personal perception of being prepared for birth, having a sense of understanding of what is happening, and having control over decision-making also appears to be related to how the birth experience is appraised and expressed.

*Subtheme 1*.*1—Being prepared for and understanding labour and birth*. The experience of childbirth may be impacted by how prepared a parent feels they were prior to labour and their level of understanding that they had about the process. Prior knowledge, as well as the ability to gather relevant information to aid understanding of unforeseen intrapartum events may impact one’s ability to navigate the challenges of the childbirth process.

*The only thing I worried about was going to the hospital maybe too soon. You have that fear of getting there and…then having the doctor tell me that I could come in tomorrow, and kind of going over him and making that decision [to go in sooner], and worrying about it being wrong… (woman) [72, p.64]*

*‘Husbands should be mentally prepared. For instance, the birthing process could be discussed before pregnancy checkups, and we should be informed about how to help our wives.’ (partner) [62, p.30]*

*‘Women sought an explanation of the basic and practical aspects of the ongoing childbirth process…nearly half of the participants were primiparous, most expressed the need for knowledge as a means of navigating the unknown.’ (review authors) [55, p.11]*

*Subtheme 1*.*2—Expectations*, *attitudes*, *and beliefs about labour and birth*. The effect of expectations, attitudes, and beliefs toward childbirth can come from the individual, or from the view point of healthcare professionals, maternity services, and models of care. Alignment of personal beliefs and attitudes with the lived experience, along with the ability to cope with changes in expectations, may influence how the birth experience is appraised.

*‘I always looked at birth as like a natural thing. I don’t like the thought of anything interfering with giving birth. It just scares me being touched or probed having to bring it on. I would rather it just go by itself. It’s a very scary thought that I have to be put on a drip and the drugs they give even though they are not harmful.’ (woman) [54, p.52]*

*‘There is a lot of built‐in anxiety especially as a first‐time mother and not knowing what to expect. Now [being induced] I have more control over my birth because I know when it will happen and I will be at the hospital when it starts.’ (woman) [79, p.406]*

*Subtheme 1*.*3 –Decision-making and control during labour and birth*. The ability to gain knowledge about birth choices, to be supported in making informed decisions, and for those choices to be respected appears to be an important concept for maintaining a sense of control during childbirth. Perceived lack of involvement in decision-making is associated with negative perceptions such as helplessness, loss of power, and reduced capability to give birth.

*‘Women described having no control over their birthing experiences. They had expected, and indeed considered it essential, that healthcare professionals would communicate information to them about the labour process, including regular updates on its status. Participants considered this pivotal to being actively involved in decisions about labour and birth, and many women reported that they were not included in the decision-making process.’ (review authors) [70, p.2145]*

*‘You can have three or more people examining you. They never ask for your permission or tell you why they must all take a turn. They do not tell you what they are feeling for or what they have found. They take you for granted because you have come to them desperate for help.’ (woman) [61, p.163]*

*‘Women felt empowered when planning their [caesarean] birth, describing being listened to, supported, informed and involved. There was a negative impact of not being heard despite indicating birthing preferences, or of not having the option to make a birth plan. As with other research, not having choice created a more clinical, surgical experience rather than “birth”.’ (review authors) [69, p.8]*

#### Dimension 2 physical aspects

This dimension includes a range of physical experiences, medical and spatial factors. Labour, birth and early postpartum are physically demanding on the body with a range of sensations and bodily functions being experienced. Whilst from a medical perspective labour is timed from the point of established labour (4cm dilated), it is recognised that for birthing women and their companions their experiences start before this as they prepare for labour, and many labour at home in the early stages. Many of the functions and sensations of labour and birth can result in discomfort or pain and in turn the use of pain relief and coping strategies. The perception of managing pain also includes aspects of the physical birth space and the care received within it. This complexity of physical experience can be further intensified by the management of the labour and birth including the broad range of labour and birth interventions that may be elected by the birthing woman or indicated by their healthcare providers, such as induction of labour or caesarean section.

*Subtheme 2*.*1—Accessing intrapartum services and experience of the labour and birth environment*. The birth experience for women and partners includes the early stages of labour prior to being admitted to the place of birth as navigating admission to intrapartum care. Findings show that activities such as monitoring the onset of labour and negotiating appropriately timed arrival at the planned place of birth are key in the birth experiences of women and partners. Likewise, the experience of the physical birthing space is important to many, for example how it affords privacy, dignity, and effective and individualised care for the birthing woman.

*‘I came to hospital thinking that I would find a safe place where I could be reassured. Instead it wasn’t like that…I didn’t feel helped, it’s more like I was abandoned.’ (woman) [57, p.78]*

*‘Some women experienced the hospital as a place to be endured that was noisy, and busy, with a lack of privacy, and too many lights, machines and strangers to allow sleep, rest and concentration on their experience… However, the hospital was also seen as a place of safety and security because of expected prompt access to HCPs and technology.’ (review authors) [64, p.25; HCP = health care providers]*

*‘During my stay in hospital all the facilities to support people with disabilities, making me feel safer and more comfortable, are important.’ (woman with visual impairment) [74, p.8]*

*Subtheme 2*.*2—Pain and its management during labour and birth*. The experience of pain is reported in terms of the type and level of pain, and the use of coping strategies and pharmacological pain relief and their relative effectiveness. For some birthing women the experience of pain and its associated management is not limited to the labour and birth but also the immediate postpartum period, e.g. related medical procedures such as suturing.

*‘I’m not into pain, I wanted an epidural. I spent most of the time screaming, “I want an epidural; I’m not doing this, I’m going home!” I gave birth completely natural with no medication whatsoever, and I was hysterical. I did not want that. I don’t like pain, and it hurts very bad, and I don’t understand why any woman would want to birth naturally’. (woman) [87, p.6]*

*‘Although the women perceived labour pain as challenging, many viewed it as playing an essential and often beneficial role in the process of childbearing and expressed a positive perception of the pain which they experienced “Its ok: this is the uterus contracting so I can meet my baby”.’ (review authors) [88, p.353]*

*‘Women described labor pain as “horrific,” “hellfire,” “awful,” a “spiraling, black abyss of pain,” and a “vortex of pain” and attributed the pain to being frightened of death…Such unbearable pain brought about comments such as “I did not know what to do” and “there was nothing I could do”.’ (review authors/women) [39, p.756]*

*Subtheme 2*.*3—Labour and birth interventions and management*. The labour and birth experience may be impacted by the physical process and outcome of labour. Those exposed to birth interventions such as induction of labour, foetal monitoring, and caesarean section have had a different physical experience compared to straightforward, physiological birth. There will be more medical procedures to experience during birth that women and partners may have limited knowledge about and be poorly prepared for.

*‘It’s like life or death. You don’t know if you’re going to live or your baby is going to live. You are not told anything. You are just rushed into theatre.’ (woman) [77, p.74]*

*‘I sort of scrambled for info from the Internet and read that the induction will be done and then repeated in 6 hours if it doesn’t work. That wasn’t actually what was done either, so it was just like we didn’t have a clue’ (woman) [79, p.407]*

*‘I always looked at birth as like a natural thing. I don’t like the thought of anything interfering with giving birth. It just scares me being touched or probed having to bring it on.’ (woman) [54, p.52]*

#### Dimension 3 emotions

In addition to the physicality of labour and birth a whole range of emotions are reported, with the emotional experience of labour and birth being unique to each person. Emotions are not static and it is reported that emotions can alter through pre labour, established labour, birth, and early postpartum and that extremes of emotions are experienced. Experiencing labour and birth can be emotionally challenging with negative feelings of fear, crisis, and a sense of failure; positive emotions are also expressed such as feeling ecstatic and empowered.

*Subtheme 3*.*1—Labour and birth as emotionally challenging*. Birth can be emotionally challenging, from awaiting the onset of labour through to birth and the early puerperium. A range of difficult emotions are apparent such as anxiety, fear, sense of failure, and lost aspirations. Not all emotional challenges are directly related to the pregnancy and childbirth as personal and social issues can affect women and potentially compound emotions that are connected to labour and birth.

*‘I was afraid because I never had surgery and uh, the life of the baby. You’re afraid for her life’. (woman) [84, p.42]*

*‘Men who acted as labour companions for their female partners may feel scared, anxious or helpless when witnessing their partners in pain during labour and childbirth.’ (review authors) [58, p.10]*

*‘Women expressed that they felt anxious and fearful about the uncertain birth process during labor. …Women also felt different emotions such as frustration, embarrassment, irritation, scared, anger, sadness, relief, and excitement during childbirth’. (review authors) [82, p.10]*

*Subtheme 3*.*2—Varied emotions in labour and birth*. The birth experience is unique to each individual and emotions change throughout the process. Extremes of feelings are reported with the ability to feel both strong positive and negative emotions within the same birth. Positive expressions of feeling powerful, empowered, and ecstatic, and negative expressions of doubt, loss of control, and feeling frightened are reported.

*‘I was so optimistic in the beginning of the latter birth… I had given birth before and I survived…so that you believe you will survive. However… I was requesting for a caesarean, I was requesting for everything! Because I just wanted to get over with it. I just said I was going to die.’ (woman) [32, p.6]*

*‘During the labour process men’s emotions fluctuated. To some extent regardless of how well labour was progressing most men worried that something might go ‘wrong’. For some these fears resulted in men constructing childbirth as ‘life-threatening’…Men found it hard to compare the moment of their child’s birth with any other experience; words such as ‘happiness’ and ‘proud’ were common.’ (review authors) [76, p.15]*

#### Dimension 4 relationships

Seldom do women labour and give birth alone and consequently the interactions and relationships with those around them influence their experience. Having a birth companion of choice can encourage personalised and humanised care. However, the attributes of that birth companion and the support that they provide will affect the experience. Interactions between healthcare professionals and birthing women are key to the birth experience.

*Subtheme 4*.*1—The importance of birth companions*. The birthing woman’s experience can be affected by the involvement and attributes of a birth companion such as a partner, co-parent, doula, or a family member. Equally, the birth companion will have their own expectations and experience of labour and birth. The importance of companion support being continuous and individualised to the needs of the birthing woman is reported.

*‘The support person was expected to be gentle, empathetic, and respectful towards the woman, knowledgeable and culturally cognisant. Other studies found that women expected a support person to be knowledgeable about childbirth, either through experience or training, for providing efficient and effective support.’ (review authors) [80, p.8]*

*‘We were a team…there were different forms of massage and…to make sure she had enough fluids and energy and moving her hips… a lot of small details easy to forget… We had written them down on a list earlier… We had prepared ourselves meticulously’. (partner) [75, p.136]*

*Subtheme 4*.*2—The influence of healthcare professionals*. The birth experience can be affected by the care received from and the interpersonal relationships with healthcare professionals. Examples of positive and negative interactions between birthing women and their companions with healthcare providers are reported, including disrespectful, dehumanised, and abusive care which is detrimental to women’s birth experiences. Level of healthcare professional support, compassion, effective communication, continuity of carer, and individualised care are reported as important features in the birth experience.

*‘I felt it was important to get close to the midwife. It was very important to me that this was a human being I could relate to and show my feelings to, whether I was happy, scared or feeling bad, that I could just show her everything’. (woman) [53, p.178]*

*‘It has to do with the people you talk to, how you feel you’re getting on, if they are listening and that. A little more humility from those I talked to would’ve been positive. I mean, when you’re in pain, you can’t take so much and you’re more irritable. It could have been my hormones of course, but it didn’t feel like that. They [midwives], of all people, should know how that can influence [the experience].’ (woman) [72, p.65]*

*‘I feel like she was rolling her eyes at me like, ugh, another one of these kids… like I wasn’t just agreeing blindly like, ‘yes you do whatever you need to do’, you know?.’ (woman) [89, p.3]*

### Subgroup analyses

Only four reviews focussed only on men/partners, making gender sub-analyses questionable. Nevertheless, an examination of all reviews where partners were included in the sample (n = 11) indicated that such reviews provided less evidence to support sub-themes of decision making and control during labour and birth; pain and its management during labour and birth; and labour and birth interventions and management. This conclusion also holds when looking only at the reviews based only on partners’ data.

Country income levels were mixed in many of the reviews, making detailed sub-analyses based on income level difficult. However, when looking at reviews with samples of low and low-middle income countries, they provided less evidence for the sub-themes of expectations, attitudes, and beliefs about labour and birth; decision making and control during labour and birth; and pain and its management in labour and birth. Conversely, such reviews provided more evidence for the sub-theme of the importance of birth companions.

## Discussion

This review aimed to synthesise reviews of qualitative studies on various aspects of the experience of childbirth in order to identify key dimensions of women and their partners’ childbirth experience and provide a comprehensive picture of this experience. The 40 reviews included spanned almost 800 studies from 81 countries in all continents. Results showed that the themes can be subsumed under four key dimensions: *Perceptions*, *Physical aspects*, *Emotions*, and *Relationships*. These are well-known aspects of the childbirth experience, each one of them in itself is not a novel finding. The main contributions of the current review of reviews are in confirming the importance of these four dimensions, identifying their 10 subthemes, and highlighting that the full picture of an individual’s birth experience includes all of these dimensions and spans the time from pre-labour through to early postpartum. Even though we reviewed reviews, comprehensive in themselves, only six of the reviews attended to all four dimensions and seven attended to three of them. Thus, over two thirds of the reviews (27/40) identified only one or two of these key dimensions. Even among the 13 reviews that focused on the general birth experience, only three attended to all four key dimensions identified here. Moreover, we identified 10 subthemes yet the most of the included reviews (35/40) each attended to only one to four of these subthemes.

As proposed by Larkin, Begley and Devane [2] in their concept analysis, our findings show that the birth experience is complex and multi-dimensional. The complexity of the birth experience is seen not only in the major role of each of the four dimensions, but also in the associations between the themes and between the different factors within each theme. For example, expectations of women and their partners interact with those of healthcare providers and affect women and partners’ perceptions, which can influence their level of preparedness before birth and their feelings of control (or not) during birth. Perceptions of pain affect women’s strategies of pain management and whether they desire or are offered pharmacological pain relief. Relationships (personal and professional) affect decision-making regarding interventions and women’s involvement in this process. The physical space may determine whether women and partners feel that their needs for privacy and dignity are addressed, and whether healthcare professionals can provide respectful care. Thus, full understanding of the birth experience requires attending to all four key dimensions *and* to the interactions between them. Importantly, what happens within and between these dimensions, happens over time along the process of labour and delivery, in a highly dynamic way.

The findings show that the birth experience does not lend itself to any simplified categorization, such as along a continuum from medical to physiological or from positive to negative. Clesse et al. [91], in their review of the evolution of birth medicalisation, noted that while this increasing trend was initially contrasted with natural conceptions of birth, nowadays medicalisation is understood as multidimensional and dynamic. Approaches such as the humanisation of birth are not solely about striving for a ‘natural’ birth’, but rather about also attending to the positive aspects of medicalization, when needed for various reasons, and about integrating biological, social, cultural, spiritual, and psychological components of childbirth. The current findings underscore the extent of individual differences in welcoming medical interventions, wishing to avoid them, or finding ways to integrate them into a personally optimal experience.

Similarly, complexity can be seen in the valence of the experience, among and within birthing persons along the birth experience. Many recent studies focussed on traumatic births, due to their long-term negative consequences. Reviews such as those reviewed here, many of which focussed on general experiences of all kinds, uncover the range and dynamic nature of both positive and negative emotions and experiences during birth. The same experience, such as pain, a medical intervention, involvement in decision-making, and so on, can be positive for one woman and negative for another. Moreover, the same woman might report a mixture of positive and negative feelings simultaneously or in different stages of birth, ranging from ecstatic to helpless.

Our review underscores several issues that have often been mentioned in the birth literature. First, the importance of (in)congruence between women’s expectations and plans formulated before birth, and their actual birth experience [18]. The current review shows that these mismatches can occur in many dimensions: From insufficient preparedness, through expectations regarding medical interventions and involvement in decisions about them, lack of control over what the staff are doing, feelings of abandonment in a setting that should have felt safe, the intensity of the pain, to disrespectful and even abusive care. Birth is unpredictable so such mismatches will exist no matter how prepared women are; the question is whether other dimensions of the experience can be attended to by providers in order to offset the negative effects of unfulfilled expectations. Second, the issue of control, whether expressed directly using this term or indirectly through experiences such as helplessness or lack of understanding of procedures and actions, is particularly important for the birth experience and underlies many of the mismatches. Third, the importance of care and support during birth. The need for respectful care and the importance of continuity in care are universal, yet the specific needs that care providers should address greatly vary among women.

There are also several issues highlighted by our review that are less often mentioned in the literature. First, the birth experience does not begin at the hospital or birthing centre and it does not end with the baby’s arrival. The physiological birth process usually begins hours before a woman or couple arrive at the place of birth. These initial hours can be quite challenging, as the couple face uncertainty, even if this is not their first birth, and need to cope and make decisions on their own. With the exception of studies and reviews that focussed specifically on early labour, less attention is given to this initial stage, despite its importance as it was found to be related to the rate of interventions later in labour [92]. Greater lack of attention was seen at the other end, right after birth–there was little mention of issues such as separation from the baby right after birth [93] or difficulties breastfeeding in the birthing room [94]. It would be interesting to expand the understanding of the birth experience along its timeline with an examination of the immediate period after the baby is born. Another not often mentioned issue that our review highlighted is the importance of the physical setting–noise, privacy, limiting entrance to the room–all these are important for creating a sense of comfort and security.

How can the knowledge summarized in the current review be used? The key dimensions differ in the ease of intervening to improve women’s birth experiences. *Perceptions* are often formed long before pregnancy [95] though they may still be somewhat malleable during women’s first pregnancy [96]. Preparing women for the uncertainty of childbirth by considering flexibility in their plans may help minimize unfulfilled expectations, and information provision during pregnancy/birth can help in coping with such mismatches, which are unavoidable. Evidence-based apps with short videos (like the Baby Buddy app in the UK, https://www.babybuddyapp.co.uk/) can be helpful [97, 98]. *Physical*, spatial and medical aspects, including navigating the initial stages and the access to intrapartum services, the birth setting, pain management and other interventions provided as much as possible in line with the woman’s wishes, can all contribute to a more positive experience. *Emotions* such as intense ups and downs of emotions can be normalized and supported with attentive and sensitive care, which connects with the *Relationships* dimension. One-to-one continuous care can provide the needed emotional support and contribute to feelings of psychological safety and empowerment. A review of interventions aimed at creating a positive perception of childbirth showed that relaxation and pain management techniques, which are often prominent in childbirth classes, are not successful strategies, while issues that stand out in our review, such as birth preparedness and readiness for complications and support during birth, are more likely to improve the birth experience [99]. Note, however, that needs vary as do levels of health literacy (so that the type of preparedness that is effective may differ across cultures for women [100] and men [101].

Using the key dimensions and subthemes identified here to devise ways to promote positive birth experiences is particularly important for women with a history of trauma, in childbirth or earlier life. A positive birth experience can be cathartic and has been found to promote relational healing in parents who have suffered previous abuse [102], healing from past traumatic birth experiences [103], as well as promoting post traumatic growth [104].

Note that the smaller number of reviews that focussed only on men or only on specific stages, issues, or modes of delivery and interventions, limited our ability to point out characteristics specific to these populations. However, the different subthemes are spread across the reviews and together provide a comprehensive universal picture. This could be used to develop instruments to assess the birth experience or to evaluate existing instruments against the key dimensions and subthemes identified here. Alongside this universal picture, it is important to also assess specific experiences, such as induction or caesarean sections.

### Strengths and limitations

The high number of 617 original studies included in 40 reviews provides a comprehensive picture of the birth experience. The smaller number of reviews that focussed on men or only on specific stages, issues, or modes of delivery and interventions, limited our ability to identify characteristics specific to these populations. Due to the paucity of research and lack of reviews we were unable to encompass issues related to single women, LGBTIQ and same-sex couples, and migrant women. Future studies can examine our findings in relation to these populations. Additionally, the main part of the analysis was led by one researcher (AD). However, a group of researchers closely critiqued it and the entire team was then invited to comment.

## Conclusions

Women give birth in many different ways in many different settings, in low, middle or high-income countries–nonetheless, what they experience as positive and as meaningful for them appears to be very similar. The birth experience from the perspective of women and partners is not solely or mainly focused on risk, as reflected in medical models [105, 106], but span a much wider perspective. Expectant parents want a safe and satisfying childbirth; yet many have concerns as they approach birth, as demonstrated by the vast literature on fear of childbirth, and by the ways that the uncertainty inherent in childbirth underlies many of the findings reported here. Preparedness in combination with attentive and sensitive care along all stages of birth, from early labour all the way to the early postpartum, appears to play a crucial role for a positive childbirth experience. Women’s and partners’ wishes regarding their childbirth and their experiences include many interdependent issues, which we subsumed under four key dimensions. These dimensions with their respective sub-themes can inform future research on the birth experience so that it covers the complexity of this experience and can be attended to by health care professionals in ways that will increase the probability of a positive childbirth experience, with its favourable sequelae.

## Supporting information

S1 Checklist PRISMA 2020 checklist. (DOCX)

S1 Table Search strategy in the four databases. (DOCX)

S2 Table Quality assessment of 33 review articles using the 21-item ENTREQ Statement and two added items. (DOCX)

S3 Table CERQual assessment of confidence. (DOCX)
