# Supplementary material for: Key dimensions of women’s and their partners’ experiences of childbirth: A systematic review of reviews of qualitative studies
Source: PLoS One. 2024 Mar 29;19(3):e0299151. doi: 10.1371/journal.pone.0299151 (PMC10980232; doi:10.1371/journal.pone.0299151)
Supplement: S1 Table — (DOCX) [file pone.0299151.s002.docx]

**Supplementary Table 1: Search strategy in the four databases.**

**Search in PubMed:**

((birth[tw] OR Births[tw] OR parturitions[tw] OR childbirth[tw] OR childbirths[tw]) OR ("labor, obstetric"[MeSH] OR labor[tw] OR labour[tw]) OR "delivery, obstetric"[MeSH] OR ("pregnancy"[MeSH Terms] OR pregnancy[tw]) OR ("parents"[MeSH Terms] OR "parents"[All Fields] OR "parent"[All Fields]) OR ("fathers"[MeSH Terms] OR "fathers"[All Fields] OR "father"[All Fields]) OR "partner"[All Fields] NOT "labor unions"[tw]) AND ((Perception[tw] OR satisfaction[tw] OR "Personal satisfaction"[Mesh] OR "patient satisfaction"[MeSH] OR birth satisfaction[All Fields]) OR birth experience[All Fields] OR ("life change events"[MeSH Terms] OR life change events[tw]) OR dissatisfaction[tw]) AND ((Meta Analysis[ti] OR Meta Synthesis[ti] OR Meta-Analysis[pt] OR Meta-ethnography[All Fields]) OR (systematic review[ti] OR systematic literature review[ti] OR systematic scoping review[ti] OR systematic narrative review[ti] OR systematic qualitative review[ti] OR systematic evidence review[ti] OR systematic quantitative review[ti] OR systematic meta-review[ti] OR systematic critical review[ti] OR systematic mixed studies review[ti] OR systematic mapping review[ti] OR systematic cochrane review[ti] OR systematic search and review[ti] OR systematic integrative review[ti]) NOT comment[pt] NOT (protocol[ti] OR protocols[ti]) NOT MEDLINE[subset] OR ("Cochrane Database Syst Rev"[Journal] AND review[pt]) OR systematic review[pt])

**Search in SCOPUS:**

(TITLE-ABS(birth* OR parturition OR childbirth* OR labor OR labour OR delivery OR pregnancy OR parent* OR father* OR partner* )) AND (TITLE-ABS(perception OR satisfaction OR dissatisfaction OR "*birth experience" OR "*birth satisfaction" AND NOT "labor unions")) AND (TITLE("meta analysis" OR "meta synthesis" OR meta-analysis OR meta-synthesis OR "meta ethnography" OR review OR "systematic review" )) AND ( LIMIT-TO ( SUBJAREA,"SOCI" ) OR LIMIT-TO ( SUBJAREA,"ARTS" ) OR LIMIT-TO ( SUBJAREA,"MEDI" ) OR LIMIT-TO ( SUBJAREA,"PSYC" ) OR LIMIT-TO ( SUBJAREA,"NURS" ) OR LIMIT-TO ( SUBJAREA,"MULT" ) OR LIMIT-TO ( SUBJAREA,"HEAL" ) OR LIMIT-TO ( SUBJAREA,"NEUR" ) OR LIMIT-TO ( SUBJAREA,"Undefined" ) ) AND ( LIMIT-TO ( DOCTYPE,"ar" ) OR LIMIT-TO ( DOCTYPE,"re" ) ) AND ( LIMIT-TO ( LANGUAGE,"English" ) )

**Search in CINAHL** (MEDLINE excluded):

( AB birth* OR AB parturition* OR AB childbirth* OR AB labor OR AB labour OR AB delivery OR AB pregnancy OR AB parent* OR AB father* OR AB partner* NOT "labor unions" ) AND ( AB perception OR AB satisfaction OR AB dissatisfaction OR AB "*birth experience" OR AB "*birth satisfaction" ) AND ( TI "meta analysis" OR TI "meta synthesis" OR TI meta-analysis OR TI metasynthesis OR TI "meta ethnography" OR TI review OR TI "systematic review" NOT TI protocol* NOT TI comment )

**Search in PsycInfo:**

( birth OR births OR parturitions OR childbirth OR childbirths OR "labor, obstetric" [mesh] OR labor OR labour OR "delivery, obstetric" [mesh] OR "pregnancy" [mesh] OR pregnancy OR parents [mesh] OR parents OR parent OR fathers [mesh] OR fathers OR father OR partner NOT "labor unions" ) AND ( perceptions OR satisfaction OR "personal satisfaction" [mesh] OR "patient satisfaction" [mesh] OR dissatisfaction OR "birth experience" OR "birth satisfaction" OR "life change events" [mesh] OR "life change events" ) AND TI ( "meta analysis" OR "meta synthesis" OR "meta-analysis" OR "meta ethnography" OR "systematic review" OR "systematic literature review" OR "systematic scoping review" OR "systematic narrative review OR "systematic qualitative review" OR "systematic evidence review" OR "systematic quantitative review" OR "systematic meta-review" OR "systematic critical review" OR "systematic mixed studies review" OR "systematic mapping review" " OR systematic cochrane review" OR "systematic search and review" OR "systematic integrative review" ) NOT PT "comment" NOT TI ( "protocol" OR "protocols" ) NOT "medline" OR Journal ( cochrane database of systematic reviews )
