# Supplementary material for: Key dimensions of women’s and their partners’ experiences of childbirth: A systematic review of reviews of qualitative studies
Source: PLoS One. 2024 Mar 29;19(3):e0299151. doi: 10.1371/journal.pone.0299151 (PMC10980232; doi:10.1371/journal.pone.0299151)
Supplement: S2 Table — (DOCX) [file pone.0299151.s003.docx]

**Supplementary Table 2: Quality assessment of the 40 review articles using the 21-item ENTREQ Statement and two added items.**

| **Items of ENTERQ and additional items** | | | | | | | | | | | | | | | | | | | | | | | | | | | | | | |
| --- | --- | --- | --- | --- | --- | --- | --- | --- | --- | --- | --- | --- | --- | --- | --- | --- | --- | --- | --- | --- | --- | --- | --- | --- | --- | --- | --- | --- | --- | --- |
| Domain: | | Introduction and methodology | | | | Literature search and selection | | | | | | | Appraisal | | | | | Synthesis of findings | | | | | | | | | Added items | | | |
|  | **Reviews** | | [**^1^**](#_heading=h.3znysh7)  ^Aim^ | [**^2^**](#_heading=h.2et92p0)  ^Synthesis methodology^ | [**^3^**](#_heading=h.tyjcwt)  ^Approach to searching^ | | [**^4^**](#_heading=h.3dy6vkm)  ^Inclusion criteria^ | [**^5^**](#_heading=h.1t3h5sf)  ^Data sources^ | [**^6^**](#_heading=h.4d34og8)  ^Electronic Search strategy^ | [**^7^**](#_heading=h.2s8eyo1)  ^Study screening methods^ | [**^8^**](#_heading=h.17dp8vu)  ^Study characteristics^ | [**^9^**](#_heading=h.3rdcrjn)  ^Study selection results^ | | [**^10^**](#_heading=h.26in1rg)  ^Rationale for appraisal^ | [**^11^**](#_heading=h.lnxbz9)  ^Appraisal items^ | [**^12^**](#_heading=h.35nkun2)  ^Appraisal process^ | [**^13^**](#_heading=h.1ksv4uv)  ^Appraisal results^ | | [**^14^**](#_heading=h.44sinio)  ^Data extraction^ | [**^15^**](#_heading=h.2jxsxqh)  ^Software^ | [**^16^**](#_heading=h.z337ya)  ^Number of reviewers^ | [**^17^**](#_heading=h.3j2qqm3)  ^Coding^ | [**^18^**](#_heading=h.1y810tw)  ^Study comparison^ | [**^19^**](#_heading=h.4i7ojhp)  ^Derivation of themes^ | [**^20^**](#_heading=h.2xcytpi)  ^Quotations^ | [**^21^**](#_heading=h.1ci93xb)  ^Synthesis output^ | | ^Pre-^  ^registration^ | ^Use of PRISMA or other Flowchart^ |  |
| **1** | Aanestad et al. 2020 | | • | • | • | | • | • | • | • | • | • | | • | • | • | • | | • | - | - | • | • | • | • | • | | - | • |  |
| **2** | Akuamoah-Boateng et al. 2018 | | • | • | • | | • | • | • | • | • | • | | • | • | • | • | | • | - | • | • | - | • | • | • | | - | • |  |
| **3** | Anderson et al. 2021 | | • | • | • | | • | • | • | • | • | • | | • | • | • | • | | • | - | • | • | • | • | - | • | | - | • | |
| **4** | Balaam et al. 2013 | | • | • | • | | • | • | • | • | • | • | | • | - | • | • | | - | - | • | • | • | • | • | • | | - | • |  |
| **5** | Beake et al. 2017 | | • | • | • | | • | • | • | • | • | • | | • | • | • | • | | • | - | • | • | • | • | • | • | | • | • |  |
| **6** | Benza et al. 2014 | | • | • | • | | • | • | • | • | • | • | | • | • | • | • | | - | - | • | • | • | • | • | • | | - | • |  |
| **7** | Bohren et al. 2015 | | • | • | • | | • | • | • | • | • | • | | • | • | • | • | | • | - | • | • | • | • | • | • | | - | • |  |
| **8** | Bohren et al. 2019 | | • | • | • | | • | • | • | • | • | • | | • | • | • | • | | • | • | • | • | • | • | • | • | | - | • |  |
| **9** | Bradley et al. 2016 | | • | • | • | | • | • | • | • | • | • | | • | • | • | • | | • | • | • | • | • | • | • | • | | • | • |  |
| **10** | Chimwaza et al. 2015 | | • | - | • | | • | • | • | • | • | • | | • | • | - | • | | - | - | - | - | - | • | • | • | | - | UNK |  |
| **11** | Clews et al. 2020 | | • | • | • | | • | • | • | • | • | • | | • | • | - | • | | - | - | - | • | • | • | • | • | | - | • |  |
| **12** | Coates et al. 2019 | | • | • | • | | • | • | • | • | • | • | | - | • | • | • | | • | • | • | • | - | • | - | • | | • | • |  |
| **13** | Coates et al. 2020 | | • | • | • | | • | • | • | • | • | • | | • | • | • | • | | • | - | • | - | - | • | • | • | | - | • |  |
| **14** | Crawford et al. 2017 | | • | • | • | | • | • | • | • | • | • | | • | • | • | • | | - | - | • | • | • | • | • | • | | • | • |  |
| **15** | Crookall et al. 2018 | | • | • | • | | • | • | • | • | • | • | | • | • | • | • | | - | - | • | • | • | • | - | • | | - | • |  |
| **16** | Crossland et al. 2020 | | • | • | • | | • | • | • | • | • | • | | • | • | • | • | | • | - | • | • | • | • | • | • | | • | • |  |
| **17** | Deys et al. 2021 | | • | • | • | | • | • | • | • | • | • | | • | • | • | • | | • | - | - | • | • | • | - | • | | - | • | |
| **18** | Elmir et al. 2010 | | • | • | • | | • | • | • | • | • | • | | • | • | - | • | | • | - | • | - | • | • | • | • | | - | • |  |
| **19** | Elmir et al. 2016 | | • | • | • | | • | • | • | • | • | • | | • | • | • | • | | • | - | • | • | • | • | • | • | | - | • |  |
| **20** | Eri et al. 2014 | | • | • | • | | • | • | • | • | • | • | | • | • | • | • | | • | - | • | - | • | • | • | • | | - | • |  |
| **21** | Fair et al. 2020 | | • | • | • | | • | • | • | • | • | • | | • | • | • | • | | • | • | • | • | • | • | • | • | | - | • |  |
| **22** | Heideveld-Gerritsen et al. 2021 | | • | • | • | | • | • | • | • | • | • | | • | • | • | • | | • | • | • | • | • | • | • | • | | • | • | |
| **23** | Hoga et al. 2013 | | • | • | • | | • | • | • | • | • | • | | • | • | • | • | | • | - | • | • | • | - | • | • | | - | • |  |
| **24** | Johansson et al. 2015 | | • | • | • | | • | • | • | • | • | • | | • | • | - | • | | • | - | • | • | • | • | • | • | | - | - |  |
| **25** | Keedle et al. 2018 | | • | • | • | | • | • | • | • | • | • | | • | • | • | • | | • | • | • | • | • | • | • | • | | - | • |  |
| **26** | Lally et al. 2008 | | • | • | • | | • | • | • | • | • | • | | • | • | - | • | | • | - | - | - | • | • | - | • | | - | - |  |
| **27** | Lou et al. 2019 | | • | • | • | | • | • | • | • | • | • | | • | • | • | • | | • | • | • | • | • | • | • | • | | - | • |  |
| **28** | Lunda et al. 2018 | | • | • | • | | • | • | • | • | • | • | | • | • | • | • | | • | - | • | • | • | • | • | • | | - | • |  |
| **29** | Mannava et al. 2015 | | • | • | • | | • | • | • | • | • | • | | - | - | - | - | | • | • | - | • | • | • | • | • | | - | • |  |
| **30** | Miyauchi et al. 2022 | | • | • | • | | • | • | • | • | • | • | | • | • | • | • | | • | • | • | • | • | • | • | • | | - | • | |
| **31** | Olza et al. 2018 | | • | • | • | | • | • | • | • | • | • | | • | • | • | • | | • | - | • | • | • | • | • | • | | • | • |  |
| **32** | Patterson et al. 2019 | | • | • | • | | • | • | • | • | • | • | | • | • | - | • | | • | - | - | • | • | • | • | • | | - | • |  |
| **33** | Puia, 2013 | | • | • | • | | • | • | • | - | • | - | | - | - | - | - | | - | - | - | • | • | • | • | • | | - | - |  |
| **34** | Sands et al. 2023 | | • | • | • | | • | • | • | • | • | • | | • | • | • | • | | • | • | • | • | • | • | - | • | | • | • | |
| **35** | Shakibazadeh et al. 2018 | | • | • | • | | • | • | • | • | • | • | | - | • | • | - | | • | - | • | • | • | • | - | • | | - | • |  |
| **36** | Shorey and Wong, 2022 | | • | • | • | | • | • | • | • | • | • | | • | • | • | • | | • | - | • | • | • | • | • | • | | - | • | |
| **37** | Thomson et al. 2019 | | • | • | • | | • | • | • | • | • | • | | • | • | • | • | | • | - | • | • | • | • | • | • | | - | • |  |
| **38** | Van der Gucht and Lewis, 2015 | | • | • | • | | • | • | • | • | • | • | | • | • | - | - | | • | - | - | • | • | • | • | • | | - | • |  |
| **39** | Watson et al. 2020 | | • | • | • | | • | • | • | • | • | • | | - | - | - | - | | - | - | - | - | - | - | • | • | | - | • | |
| **40** | Wigert et al. 2020 | | • | • | • | | • | • | • | • | • | • | | • | • | • | • | | • | - | • | • | • | • | • | • | | - | • |  |

*Note*s. (•), ENTREQ item was reported by the corresponding article; (-) not stated or not applicable. UNK: Unknown shows that Figure 1 which was related to these items was not available; *Watson et al. is a scoping review and therefore did not appraise the quality of the reviewed studies.

The last two columns are not part of the ENTREQ items, they were added for the current review and therefore are separated with a thicker vertical line. They relate to the use of any pre-registration procedure and the use of PRISMA or any flowchart toe describe the search and screening results.
