# Supplementary material for: Key dimensions of women’s and their partners’ experiences of childbirth: A systematic review of reviews of qualitative studies
Source: PLoS One. 2024 Mar 29;19(3):e0299151. doi: 10.1371/journal.pone.0299151 (PMC10980232; doi:10.1371/journal.pone.0299151)
Supplement: S3 Table — (DOCX) [file pone.0299151.s004.docx]

**Supplementary Table 3: CERQual Assessment of Confidence**

|  | **Sub-Themes** | **CERQual Assessment** | | | | | | |
| --- | --- | --- | --- | --- | --- | --- | --- | --- |
|  |  | ***Methodological Limitations*** | ***Relevance*** | ***Coherence*** | ***Adequacy*** | ***CERQual Assessment of Confidence*** | ***Explanation of CERQual Assessment of Confidence*** |  |
| **Theme 1 - Perception** | ***Being Prepared for and Understanding Labour and Birth*** | No or Very Minor Concerns  Chimwaza et al., 2015 is a supporting review which has a low ENTREQ score of 14. | No or Very Minor Concerns | Minor Concerns  As 6 studies (Akuamoah-Boateng & Spencer, 2018; Clews et al., 2020; Crookall et al., 2018; Crossland et al., 2020; Deys et al., 2021; Heidefeld-Gerritsen et al., 2021) report on the specific issues of induction; water birth; perineal trauma; assisted delivery; caesarean birth; and care experiences of women with physical disabilities. | Minor Concerns  As 3 of the 12 supporting reviews had small samples (5 or fewer studies). | Moderate Confidence | No or very minor concerns over methodological limitations and relevance. Minor concerns over coherence and adequacy owing to narrow focus and small sample size of included reviews. |  |
|  | ***Expectations, Attitudes, and Beliefs about Labour and Birth*** | No or Very Minor Concerns | No or Very Minor Concerns | Minor Concerns  As 2 studies (Akuamoah-Boateng & Spencer, 2018; Lou et al., 2019) report on the specific issue of Induction and 1 on caesarean birth (Deys et al., 2021) | Minor Concerns  As 2 of the 12 supporting reviews had small samples (5 or fewer reviews). | Moderate Confidence | No or very minor concerns over methodological limitations and relevance. Minor concerns over coherence and adequacy owing to narrow focus and small sample size of included reviews. |  |
|  | ***Decision-making and Control During Labour and Birth*** | No or Very Minor Concerns  Two of the 13 supporting reviews have low ENTREQ scores (Watson et al., 2021, a score of 11; Puia, 2013, a score of 12). | No or Very Minor Concerns | Minor Concerns  As 2 studies (Akuamoah-Boateng & Spencer, 2018; Lou et al., 2019) report on the specific issue of Induction. | Minor Concerns  As 3 of the 13 supporting reviews had small samples (5 or fewer studies). | Moderate Confidence | No or very minor concerns over methodological limitations and relevance. Minor concerns over coherence and adequacy owing to narrow focus and small sample size of included reviews. |  |
| **Theme 2 – Physical aspects** | ***Accessing Intrapartum Services and Experience of the Labour and Birth Environment*** | No or Very Minor Concerns  Chimwaza et al., 2015 is a supporting review which has a low ENTREQ score of 14. | No or Very Minor Concerns | Minor Concerns  2 of the 10 reviews (Heidefeld-Gerritsen et al., 2021; Sands et al. 2023) reported on the specific issues of care experiences of women with physical disabilities and birth environments for women with complex pregnancies, respectively. | No or Very Minor Concerns  As there are 10 reviews supporting this sub-theme and 1 had a small sample (5 or fewer studies). | Moderate Confidence | No or very minor concerns over methodological limitations, relevance, and adequacy. Minor concerns over coherence as 2 reviews had a narrow focus. |  |
|  | ***Pain and its Management During Labour and Birth*** | No or Very Minor Concerns  Puia, 2013 is a supporting review which has a low ENTREQ score of 12. | No or Very Minor Concerns | Minor Concerns  As 3 of the 10 supporting reviews are looking at specific issues (Clews et al., 2020; Crookall et al., 2018; Puia, 2013) of water birth, perineal suturing and c-section. | No or Very Minor Concerns  1 or the 10 supporting reviews had a small sample (5 or fewer studies). | Moderate Confidence | No or very minor concerns over methodological limitations,relevance and adequacy. Minor concerns over coherence owing to 3 reviews having a narrow focus. |  |
|  | ***Labour and Birth Interventions and Management*** | No or Very Minor Concerns | No or Very Minor Concerns | Minor Concerns  As 2 of the 5 supporting reviews (Akuamoah-Boateng & Spencer, 2018; Lou et al., 2019) examined the specific issue of induction and 1 review (Crawford et al., 2017) examined fetal monitoring. However, this is less of a concern than in other sub-themes as the focus here is on interventions. | Minor Concerns  As there are only 5 reviews supporting this sub-theme of which 2 had small samples (5 or fewer studies). | Moderate Confidence | No or very minor concerns over methodological limitations and relevance. Minor concerns over coherence and adequacy owing to narrow focus and small sample size of included reviews. |  |
| **Theme 3 – Emotions** | ***Labour and Birth as Emotionally Challenging*** | No or Very Minor Concerns  Puia, 2013 is a supporting review which has a low ENTREQ score of 12. | No or Very Minor Concerns | No or Very Minor Concerns | No or Very Minor Concerns  As 2 of the 15 reviews supporting this sub-theme of had a small sample (5 or fewer reviews). | High Confidence | No or very minor concerns over methodological limitations, relevance, adequacy, and coherence. |  |
|  | ***Varied Emotions in Labour and Birth*** | No or Very Minor Concerns  Chimwaza et al., 2015 is a supporting review which has a low ENTREQ score of 14. | No or Very Minor Concerns | No or Very Minor Concerns | Minor Concerns  As there are only 7 reviews supporting this sub-theme of which 2 had small samples (5 or fewer studies). | High Confidence | No or very minor concerns over methodological limitations, relevance, and coherence. Minor concerns over adequacy due to there being only 7 supporting reviews, of which 4 had a small sample. |  |
| **Theme 4 - Relationships** | ***The Importance of Birth Companions*** | No or Very Minor Concerns  Chimwaza et al., 2015 is a supporting review which has a low ENTREQ score of 14. | No or Very Minor Concerns | No or Very Minor Concerns | No or Very Minor Concerns | High Confidence | No or very minor concerns over methodological limitations, relevance, coherence, and adequacy. |  |
|  | ***The Influence of Healthcare Professionals*** | No or Very Minor Concerns  Two of the 22 supporting reviews have low ENTREQ scores. Watson et al., 2021 a score of 11 and Puia, 2013 a score of 12. | No or Very Minor Concerns | No or Very Minor Concerns  One of the 22 supporting reviews (Heidefeld-Gerritsen et al., 2021) reported on the specific issue of care experiences of women with physical disabilities. | No or Very Minor Concerns  1 of the 22 supporting reviews (Watson et al.,2021) had a small sample size of 5 studies. | High Confidence | No or very minor concerns over methodological limitations, relevance, coherence, or adequacy. One of the reviews (Watson et al., 2021 has a low ENTREQ score, low sample size (5) and focused on a niche population. However, there are a further 21 reviews supporting this theme. |  |
